# "Recommends" for Insured Persons

**Published:** 1920-03-06

**Authors:** 


### "Recommends" for Insured Persons.

### A NORTHAMPTON DECISION.

Mr. C. S. Risbee, Secretary-Superintendent of Northampton General Hospital, has issued on behalf of his Committee the following statement to insured persons:

On and after March 1 next, persons who are insured under the National Insurance Act will only be admitted for out-patient treatment at this hospital upon the written advice of their panel doctor, in addition to which it will be necessary for them to bring a subscriber's "letter of recommendation."

Should an insured patient attend without bringing such written advice, he or she will be examined by one of the resident medical officers, and, if not found to be an urgent case, will be referred to the panel doctor to obtain a written letter. In all cases where treatment at the hospital is not considered necessary the subscribers' letters of recommendation will be returned.

Accidents and emergency cases are not affected: they will receive immediate attention as heretofore.

February, 1920.

